# Harmful Alcohol Use and Associated Socio-Structural Factors among Female Sex Workers Initiating HIV Pre-Exposure Prophylaxis in Dar es Salaam, Tanzania

**DOI:** 10.3390/ijerph20010698

**Published:** 2022-12-30

**Authors:** Hanne Ochieng Lichtwarck, Method Rwelengera Kazaura, Kåre Moen, Elia John Mmbaga

**Affiliations:** 1 Department of Community Medicine and Global Health, Institute of Health and Society, Faculty of Medicine, University of Oslo, 0450 Oslo, Norway; 2 Department of Epidemiology and Biostatistics, Muhimbili University of Health and Allied Sciences, Dar es Salaam 11103, Tanzania

**Keywords:** social epidemiology, sex work-related mobility, criminalization, alcohol-related disorders, gender-based violence, Sub-Saharan Africa, substance abuse, sex work, key population

## Abstract

Harmful alcohol use is an important risk factor for premature mortality and morbidity and associated with increased HIV risk and lower uptake of and adherence to HIV interventions. This study aimed to assess the extent of harmful alcohol use and associated socio-structural vulnerability factors among female sex workers in Dar es Salaam, Tanzania, a key population in the HIV epidemic. Data from a study of female sex workers initiating pre-exposure prophylaxis (PrEP) recruited through respondent driven sampling were used. We assessed harmful alcohol use with the Alcohol Use Disorders Identification Test (AUDIT) defined as having an AUDIT score ≥ 16. Associations between harmful alcohol use and socio-structural factors were assessed using logistic regression with marginal standardization. Of the 470 women recruited, more than one third (37.3%) had a drinking pattern suggestive of harmful alcohol use. Such use was independently associated with sex work-related mobility (aPR: 1.36, 95% CI: 1.11–1.61), arrest/incarceration (aPR: 1.55, 95% CI: 1.27–1.84) and gender-based violence (aPR: 1.31, 95% CI: 1.06–1.56). The high prevalence of harmful alcohol use and the interconnectedness with socio-structural factors indicate a need for a holistic programmatic approach to health for female sex workers. Programming should not solely direct attention to individual behavior but also include strategies aiming to address socio-structural vulnerabilities.

## 1. Introduction

Alcohol use is one of the leading risk factors for premature death and disability worldwide [1]. Three million people die each year from alcohol-related causes, and alcohol is responsible for 13.5% of all deaths among adults aged between 20 and 39 years [1]. The African continent carries the greatest burden of disease and injuries related to alcohol and has among the highest occurrences of heavy drinking among those who consume alcohol [1].

Social and cultural norms, as well as policies of various kinds, influence the use of alcohol, and those in lower socioeconomic positions experience more harm than those more affluent at the same level of consumption [2]. Among female sex workers in Sub-Saharan Africa (SSA), alcohol consumption is common [3,4]. Much commercial sex work takes place in establishments serving alcohol [3,5,6,7,8,9], and alcohol use can facilitate transition into and maintenance of sex work [3]. In Tanzania, research has found high rates of alcohol consumption among female sex workers [10,11]. In one study from Dar es Salaam, the country’s largest city, more than 28% reported drinking more than four times a week, while nearly half (48%) consumed 5–6 units of alcohol or more on a typical day [12]. Furthermore, it is estimated that approximately 15% of women selling sex in Dar es Salaam, are HIV positive [12]. Alcohol use has been associated with unprotected sex and HIV infection [1,13,14,15,16,17,18,19,20,21]. Moreover, drinking has the potential to exacerbate the course of HIV [15]. Alcohol has also been associated with poor uptake of, and lower adherence to, various HIV interventions [22,23,24].

While individual and behavioral determinants of alcohol use have been studied to some extent in SSA, socio-structural factors have received limited attention. Socio-ecological frameworks [25] can aid in the identification of socio-structural factors that may impact alcohol use among female sex workers. These frameworks do not limit themselves to focus on individual characteristics that put people at risk at the micro level but illustrate how the individual is situated within different layers of influence: from the individual level, via the interpersonal, community, and institutional levels, to the macro/structural level, and how these interact and shape health outcomes. Shannon et al. [26] have further developed a HIV structural determinants framework for female sex workers. Within this framework, macro-structural determinants, such as short-term mobility to sex work “hotspots” and the effects of criminalizing policies are taken into consideration. Additionally, determinants at the community and interpersonal levels, such as gender-based violence (GBV), stigma and social support, are highlighted. A few empirical studies from SSA have indeed found an association between GBV against female sex workers and alcohol use [6,9,27,28], but apart from this, studies into the relationships between socio-structural factors and alcohol use among female sex workers in Africa are scarce.

To achieve the global prevention and treatment goals in connection with harmful alcohol use [29,30], investigating potential links between socio-structural factors and harmful alcohol use among HIV at-risk populations is important. Failing to address these factors may render prevention efforts largely ineffective. Therefore, in this study, we aimed to assess associations between some potential socio-structural determinants (mobility, incarceration, GBV, stigma and social support) and harmful alcohol use among female sex workers in Tanzania. Additionally, for the first time within this population in Tanzania, we sought to estimate the extent of harmful alcohol use using the standard composite measure Alcohol Use Disorders Identification Test (AUDIT) [31].

## 2. Materials and Methods

### 2.1. Study Design and Participants

This paper presents findings from a baseline survey carried out as part of a quasi-experimental study evaluating the effectiveness of a mobile phone application in improving adherence to PrEP among female sex workers in Dar es Salaam, Tanzania. The city has a population of more than five million [32], and an HIV prevalence of 6.3% among women [33]. Being a metropolitan city, Dar es Salaam has one of the largest concentrations of female sex workers in the country, estimated to be around 9500 [12,34].

Female sex workers were defined as women who had exchanged sex for money or goods during the last three months prior to recruitment. Participants were eligible if they were 18 years or older, had lived in Dar es Salaam for at least 6 months, owned a smartphone, and were eligible for PrEP. The eligibility criteria for PrEP initiation were being HIV seronegative, being at substantial risk of HIV infection with no suspicion of acute HIV infection, having serum creatinine clearance > 60 ml/min and being willing to consent for and use PrEP as prescribed [35].

### 2.2. Procedures

In collaboration with a local clinic, we recruited participants using respondent-driven sampling (RDS) [36]. RDS is a modified “snowball-sampling” technique in which participants recruit from their own network, but unlike in snowball-sampling, each recruit must give an estimate of their network size. This information is used to calculate probability weights to account for the non-random nature of the sampling with the aim of arriving at a more representative sample [36]. We selected nine initial study participants (so-called “seeds”) with diverse characteristics. Each seed was given three recruitment coupons and encouraged to invite other female sex workers to take part in the study. These were in turn asked to invite yet other participants, thus setting off a process that led to several waves of recruitment. Recruitment continued until the desired sample size had been achieved. Participants received 8000 Tanzanian Shillings (≈4 USD) as compensation for their transportation costs and the time spent participating in the study, and 4000 TZS (≈2 USD) for each new study participant they recruited.

With regard to the analysis in this paper, a sample size of 383 would be required based on a single proportion sample size calculation with a confidence level of 95%, a 5% margin of error, and estimated 50% of participants drinking excessively [37]. The mother study final recruited sample was 470 women.

After being assessed for eligibility and consenting to study participation, trained research assistants interviewed participants face to face during their first clinic visit, using a survey questionnaire prepared as part of the mother-study. The questionnaire had been developed in English and translated to Swahili by Swahili-speaking members of the team. The translation was quality assured by the other researchers and pre-tested among research assistants and members of the sex worker community to assure that questions were understood and captured the intended meaning. Before the survey started, we had installed the questionnaire into “Nettskjema”, an online tool for conducting surveys, developed by the University of Oslo [38]. It enabled the research assistants to directly plot respondents’ answers into the questionnaire solution while conducting the interviews using hand-held tablets. Data were encrypted and directly transferred to a highly safe storage platform, TSD [39]. This process limited the use of pen and paper, which was only needed during the screening and consent process and by the clinic for their medical records. The recruitment of study participants and baseline data collection took place between March and July 2021.

### 2.3. Instruments and Measures 

#### 2.3.1. The Survey Questionnaire

The survey questionnaire contained 13 sections and covered topics assessed to be of relevance for PrEP use. It was developed based on a review of literature and informed by socio-ecological frameworks [25] and previous studies among key populations conducted by members of the research team. Questionnaire items included were alcohol consumption, drug use, socio-demographic characteristics, sex work history, sexual practices, socio-structural determinants, HIV knowledge, access to and satisfaction with health services, PrEP motivation, behavioral skills and self-efficacy, and questions relating to mobile health. We used previously validated scales where available, such as AUDIT to measure alcohol use [31], the female sex worker stigma scale [37], PHQ2 and GAD-2 assessing mental distress [40,41], the Duke UNC-Functional Social Support Questionnaire to measure social support [42], and a PrEP stigma scale [43]. Details related to items relevant for this analysis are presented below.

#### 2.3.2. Outcome: Alcohol Use Assessment

Alcohol use was assessed using the “Alcohol Use Disorders Identification Test” (AUDIT) [31]. This 10-item measure has been developed by the WHO to aid health practitioners in identifying people who could benefit from interventions aiming at reduced alcohol consumption. We used a Swahili version which has been validated and has displayed good psychometric properties among traumatic brain injury patients in Tanzania [44], with only minor modifications in wording. The instrument screens for hazardous and harmful alcohol use and alcohol dependency. The three first questions relate to hazardous drinking, defined as drinking that increases the risk of harmful consequences although the user has no sign of any disorder. The questions inquire about drinking frequency, typical amount of alcohol intake, and frequency of heavy drinking. Four questions in the tool are specifically aiming to capture harmful alcohol use and includes questions on blackouts, guilt, injuries, and other people’s concern about the person’s alcohol intake. Furthermore, the instrument elicits information on typical dependency signs, such as increased salience of drinking, impaired control over drinking, and morning drinking. The composite score ranges from 0 to 40. WHO suggests that scores between 8 and 15 be categorized as hazardous use, scores between 16 and 19 as harmful use, and scores above 20 as likely dependency [45]. In this study we used a cut-off score of 16, as this would estimate the proportion of women with either a harmful pattern of alcohol use or likely dependency. Individuals in both categories are in need of counselling, monitoring and/or treatment as per the WHO guideline [45]. Women who reported no drinking during the past year did not complete the AUDIT questionnaire since their total score would not exceed 6, and they were automatically included in the low-risk group without further assessment. In addition to the AUDIT questions, we also asked a question about drinking while doing sex work. This was done to capture context specific drinking related to the women’s occupation. 

#### 2.3.3. Socio-Structural Factors and Other Covariates

The questionnaire further included questions aiming to elicit socio-structural vulnerability: gender-based violence, arrest/incarceration, short-term mobility, stigma, and low social support. The first three of these domains were assessed using single item questions with dichotomous response categories (yes/no). In the analysis, we merged two questions (on sexual assault and on violence, respectively) to create a single variable for gender-based violence. The questions were “Have you experienced physical violence (like being beaten) during the last 12 months?” and “Have you been forced to have sex during the last six months?” If participants confirmed to either, they were asked who the perpetuator(s) were. To assess mobility participants were asked: “Within the last 6 months have you ever travelled to another city or county to perform sex work?” Finally, we measured experiences with law enforcement with the question: “Have you been arrested by the police the last 12 months?” If the participants confirmed, we also inquired about the nature of the offence. 

Stigma related to sex work was assessed using a 13-item, four-point Likert scale previously validated by research teams working with female sex workers in the Dominican Republic and Tanzania [37] The development of this measure has been informed by the HIV Berger Stigma Scale [46] and adapted to assess aspects of sex work stigma. The FSW stigma scale included statements such as “It’s easier to avoid friendships than worry about telling others you are a sex worker” with answer options ranging from “strongly agree” to “strongly disagree”. The higher the score, the more stigma (range 13–52). 

We used an eight statement five-point Likert scale, the Duke UNC-Functional Social Support Questionnaire [42], to measure social support. The scale was originally developed in the US, but has later been tested in Rwanda and has demonstrated good convergent and discriminant validity among HIV positive men and women [47]. It includes statements such as “I have people who cares about what happens to me” and “I get chances to talk to someone I trust about my personal or family problems”. Answer options ranged from “Much less than I would like” to “As much as I would like”, with higher scores representing increased social support (range 1–5). The FSW stigma scale and the social support scale were dichotomized at the median. Reliability coefficients for the scales were good, both at 0.88.

Other variables of interest for this analysis were sociodemographic factors and factors related to sex work such as the number of years that had passed since the person sold sex for the first time, the number of clients per month, whether the person had any other work, and any drug use. These circumstances were hypothesized to be related to the socio-structural exposures and harmful alcohol use.

### 2.4. Data Analysis

We estimated proportions for categorical variables, while medians with interquartile ranges (IQR) were calculated for continuous variables. When using RDS recruitment, participants with larger networks are more likely to be overrepresented in the sample. Weights were thus calculated as the inverse of a participants’ network size and used when estimating proportions [36]. We performed Chi-square tests to assess associations between harmful alcohol use (AUDIT ≥ 16) and socio-demographic and socio-structural factors. To estimate independent effects of the three socio-structural exposures found significant in the bivariate analyses, we built three separate models with harmful alcohol use as the outcome.

This analytic strategy avoids “the table 2 fallacy” [48] where two or more coefficients in one single multivariable model are interpreted as estimates for meaningful effects. It allows adjusting for different potential confounders for separate exposure-outcome pairs. To aid in the decision of which variables to adjust for, we drew three directed acyclic graphs (DAGs) using the software tool “DAGitty” [49], one for each of the three exposures (GBV, mobility and arrest incarceration) and harmful alcohol use. In the final models, we adjusted for variables with p-values less than 0.2 in bivariate analysis, as well as for age. When drawing the DAGs, we were guided by socio-ecological theory, former research, and plausible assumptions about relationships between the variables. Given that the outcome of interest was common and the odds ratio (OR) would thus have overestimated the relative risk (RR), we used logistic regression and consecutively marginal standardization to compute prevalence ratios (PR) and adjusted prevalence ratios (aPR) [50]. The analyses were conducted using unweighted data as there is no consensus on how to best perform analyses of associations between two variables with RDS data [36,51]. Studies that have compared the use of weighted and unweighted regression have found that weighted regression can introduce bias and high type 1 error rates [51,52], partly due to the dependence on reported network size. Unweighted regression has been found to perform better using both simulated and real-life data suggesting that this is the preferable approach [51,52]. All analysis were two-tailed, and the significance level was set at 5%. We used STATA/SE 16.0 (StataCorp2019, College Station, TX, USA) for all analysis. 

### 2.5. Ethical Considerations

The study received ethical clearance from the Ethical Review Committee of the Muhimbili University of Health and Allied Sciences (MUHAS), the Tanzania National Institute of Medical Research (NIMR), and the Regional Committee for Medical and Health Research Ethics (REK) in Norway. Foreign investigators received a research permit from the Tanzania Commission for Science and Technology (COSTECH). All participants provided written consent to take part in the study after being informed about the study’s aims and procedures. 

## 3. Results

### 3.1. Socio-Demographic and Sex Work-Related Characteristics

A total of 470 participants with a median age of 25 years (IQR: 22–29) were recruited. Table 1 presents the distribution of their socio-demographic and socio-structural characteristics. Three quarters (361; 75.8%) had never been married and 442 (90.2%) reported to have completed primary education. The median monthly income from sex work was 300,000 Tanzanian shillings (≈130 USD). Nearly two thirds (283; 59.4%) of the participants did not engage in any other income-generating work (or studies) besides sex work. About half (255; 46.2%) had started sex work five or more years earlier. The median number of clients (vaginal sex) per month was 15 (IQR: 6–30).

### 3.2. Socio-Structural Factors

The median female sex worker stigma score was 31 (IQR: 27–35), whereas the median social support score was 3.4 (IQR: 2.5–4.5). Overall, 241 (43.7%) of the women had experienced GBV during the past 12 months, and the most frequent perpetrators had been sex work clients. One in five (126; 20.6%) had been arrested by the police in the same period. Of these, 75 (54.7%) had been arrested for selling sex. A total of 179 (28.4 %) participants had travelled to another city or country to do sex work in the six months-period preceding recruitment.

### 3.3. Alcohol and Drug Use

As Table 2 illustrates, 405 (86.2%) participants reported that they had used alcohol in the year preceding the survey. Of these, 360 (69.6%) had an AUDIT score of 8 or above (indicative of “hazardous alcohol use”) while 230 (37.3%) had an AUDIT score of 16 or more (suggestive of “harmful alcohol use” or “alcohol dependency”). More than half of the whole sample (296; 53.6%) had been drinking alcohol the last time they had sex with a client.

**Figure 1:**
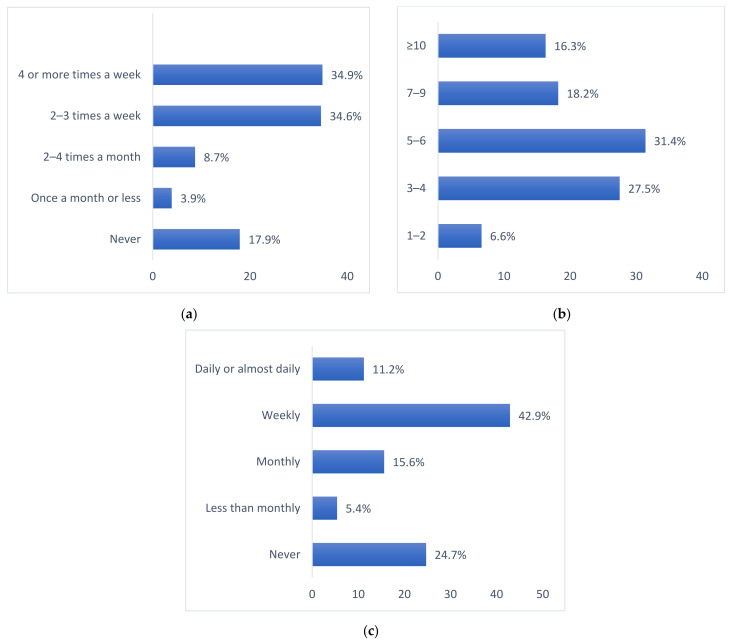
AUDIT C. Proportions are weighted: (**a**) Distributions of reported frequency of drinking alcohol among all participants (n = 470); (**b**) Distributions of reported number of drinks on a typical day when drinking among participants reporting drinking the previous year (n = 405); (c) Distributions of reported frequency of “binge drinking” (drinking ≥ 6 units of alcohol on one occasion) among participants reporting drinking the previous year (n = 405).

To allow for comparison with other studies, we present the distribution of responses for three of the AUDIT questions in Figure 1a–c. These questions make up the shorter version of AUDIT, called AUDIT-C [53].

### 3.4. Factors Associated with Harmful Alcohol Use

Arrest/incarceration, GBV and sex work-related mobility were all significantly associated with harmful alcohol use (AUDIT ≥ 16) in bivariate analysis (Table 3). Additionally, the number of years since starting sex work, the number of clients per month, not having other work than sex work, and having ever used drugs were also significantly associated with harmful alcohol use (Table 3).

### 3.5. Socio-Structural Factors Independently Associated with Harmful Alcohol Use

The results from the bivariate and the multivariable regression models of the association between socio-structural factors and harmful alcohol use are presented in Table 4. We found that participants with a history of arrest/incarceration during the past 12 months had a 55% higher prevalence of harmful drinking than those who had not been incarcerated (aPR-adjusted prevalence ratio: 1.55, 95% CI: 1.27, 1.84). Female sex workers who had experienced GBV during the past 12 months had an aPR of 1.31 (95% CI: 1.06–1.56) compared to those who had not. Moreover, mobility to another city for sex work was independently associated with a 36% increased prevalence of harmful alcohol use as compared to participants who had not travelled (aPR, 1.36. 95% CI: 1.11–1.61).

## 4. Discussion

We found that harmful alcohol use was prevalent among female sex workers initiating pre-exposure prophylaxis in Dar es Salaam, Tanzania. Three socio-structural factors, namely sex work-related mobility, arrest, and gender-based violence, were independently associated with harmful alcohol use, suggesting an intersection of vulnerabilities.

The proportion of study participants engaging in harmful alcohol use was high compared to what has been reported from the neighboring countries of Kenya, Malawi, and Uganda, where AUDIT scores have ranged from 9.5% to 27% among female sex workers [17,54,55]. The higher prevalence in our sample is consistent with the higher overall alcohol use in Tanzania (9.4 L per capita per year) compared to that of Kenya (3.4 L) and Malawi (3.7 L) [1]. Ugandans, however, have equally high alcohol consumption as Tanzanians [1]. 

Female sex workers are confronted with multiple health risks. Studies indicate that female sex workers in diverse settings are disproportionally affected by violence [56], sexually transmitted infections [26,57] and adverse mental health outcomes [58]. Harmful alcohol use with its social and health-related consequences adds to this burden. As alcohol use can increase HIV risk, it has the potential to maintain or exacerbate already existing inequalities within the HIV epidemic. Moreover, a recent study found that alcohol use was associated with non-adherence to PrEP [24]. This finding, combined with the high prevalence of harmful alcohol drinking in our study and other studies, suggest that incorporating alcohol screening, counseling, and treatment in PrEP provision in Tanzania and similar settings might be warranted.

With alcohol use disorders known to be impacted by societal factors, it is however not likely that individual-oriented strategies to reduce alcohol consumption will be sufficient [59,60]. Epidemiological HIV prevention research typically focuses on individual risk, and in the literature on HIV and female sex workers, alcohol use is usually referred to as a “behavioral risk factor”. This body of research tells an incomplete story by placing the main responsibility on the individual whilst ignoring interpersonal, organizational, community and public policy factors that take part in shaping these risks. We therefore sough to explore some of this socio-structural vulnerability and its relation to harmful alcohol use by focusing on socio-structural factors which have been identified as important determinants for HIV among female sex workers [26]. 

In our sample, about half of the participants had experienced GBV and this was associated with a 31% increased prevalence of harmful alcohol use. An association between violence and problem drinking has previously been established [61], and earlier research among women engaging in transactional sex in SSA, including in Tanzania, has also found such a relationship [6,9,17,27,28,62,63]. Few previous studies have, however, used composite measures such as AUDIT to investigate the association with harmful alcohol use in a more comprehensive way. Qualitative research from Tanzania has explored how aspects of sex work, such as alcohol use, facilitates risks for HIV and gender-based violence [64]. They found that this occurred in interactions with clients at three moments in time: when meeting clients at the bar, where alcohol could increase the risk for violence; during negotiations, when clients could become violent if a sex worker refused sex after drinking alcohol purchased by the prospective client; and during sex, when alcohol intake could render the women unconscious and facilitate unwanted or unprotected sex [64]. Other studies, especially from high-income countries, demonstrate how the relationship between alcohol use and GBV may also work in the opposite direction, i.e., gender-based violence can lead to problematic drinking [61,65,66]. The effect measured in cross-sectional studies (like ours) is likely a consequence of both these relationships.

About a quarter of the participants in this study reported to have been arrested during the past 12 months preceding the survey and the most common reason cited was sex work (60%). We found that women who had been arrested were 55 % more likely to report harmful alcohol use than those who had not. Criminalization of sex work has been linked to poorer health-related outcomes among female sex workers, including increased HIV risk and higher levels of inconsistent condom use, stigma, and food and income instability [26]. Contact with law enforcement, such as arrests, has, as shown in studies from high income settings, the potential to inflict physical and psychological ill health [67,68]. It is possible that stress processes that partly explain the effect of arrests/incarceration on depression and anxiety [68] could also influence alcohol consumption. It is however also plausible that part of the association can be explained by the increased risk of getting arrested that is experienced by people engaging in harmful alcohol use.

The third socio-structural factor we found to be associated with harmful alcohol use, was sex work-related mobility. Women who had moved for sex work during the past 6 months had a 36% higher relative likelihood of engaging in harmful alcohol use than those who had not been mobile. Mobility has been linked to both positive and negative health outcomes for sex workers [69]. Typically, intra-urban and intra-district mobility and short-term movement to so-called “hotspots” for sex work has been shown to increase HIV vulnerability [69]. In this study, mobility was common, with over two thirds of the participants having moved for sex work during the past 6 months. Mobility can, for some, reflect social and economic instability. One the other hand, sex workers who temporarily find themselves away from known environments may receive less support from their social networks and for that reason experience added vulnerability to outer stressors. Mobility has for instance been found to be a risk factor for gender-based violence among female sex workers in Iringa, Tanzania [70,71]. With regard to the relationship between mobility and alcohol use, a study from India by Saggurti et al. [72] with over 5000 mobile sex workers, found that mobility was associated with alcohol consumption before sex. There is however a need for more longitudinal quantitative studies to measure the direction of effect and for qualitative studies to explore this relationship among sex workers. 

The findings of this study have implications for how alcohol use practices should be addressed and suggest that public health would benefit from engaging with socio-structural factors identified in this study. Individualized behavior change strategies can be effective, at least in the short-term [27], but are likely to be insufficient alone [73]. Community empowerment approaches addressing socio-structural challenges such as GBV and social cohesion among female sex workers in Tanzania have proven effective, both for HIV prevention and treatment outcomes and for reduced alcohol consumption [74]. Similar interventions could be expanded and tested. Additionally, efforts to improve female sex workers access to care while travelling, have also been suggested previously [70]. The interventions should be flexible and allow for prevention and treatment seeking at multiple locations regardless of city of origin. Finally, structural interventions and policies aiming to ameliorate the life circumstances of female sex workers by reducing their exposure to arrest and incarceration will likely be of impact. It should also be noted that alcohol specific structural level interventions, such as restrictions on availability of alcohol, alcohol advertising and alcohol taxations have only to a limited degree been utilized in SSA including in Tanzania [1] and might represent effective policy approaches. It is however unclear if such restrictions will have the anticipated effect among a group such as female sex workers, without addressing other co-occurring structural vulnerabilities such as those highlighted in this paper.

The results presented in this study should be interpreted considering some limitations. Firstly, the cross-sectional nature of the study limits our ability to draw causal inferences. Our aim was however to assess whether relationships were present in exploratory analyses as a way of directing attention to what we expected would be overlapping risks confronted by female sex workers and discuss how these relationships might be understood. With regard to the independent variables, our study did not distinguish between short term arrest/detention and longer in-prison sentences, nor between short and long-term mobility in connection with sex work. Future research should expand on these categories to elucidate these relationships further. We did not find statistically significant relationships between female sex work stigma and social support, and harmful alcohol use. Alternate analyses using the summary scores as continuous variables instead of categorical exposures did not change this conclusion. It is possible that the lack of an association between social support and harmful alcohol use could be attributed to drinking practices promoting social contact with other sex workers within drinking venues. This could have neutralized any negative effect that social support might have had. We found it surprising that female sex work stigma was not associated with harmful alcohol use. One possible explanation could be that women with higher stigma, as a response, would limit visits to bars or other avenues where they could be stigmatized, thus also limiting alcohol intake. We furthermore acknowledge that our study participants, who were enrolled in a PrEP program and a mHealth study, may not be representative of all female sex workers in Tanzania. Given the challenges related to the execution of RDS and the fact that this sampling approach does not equate to random sampling, we have presented both unweighted and weighted proportions to illustrate the differences in some of the estimates. As in all studies on topics that may be perceived to be sensitive, desirability bias might have affected the way people answered questions. Despite the mentioned limitations, the study provides important new knowledge about a significant health challenge facing a population with several health-related vulnerabilities.

## 5. Conclusions

Our study supports earlier research that has documented that problematic alcohol use is common among female sex workers. To our knowledge, it is the first study conducted among female sex workers in Tanzania that has used the AUDIT to assess harmful alcohol use. The demonstrated interconnectedness between socio-structural factors and harmful alcohol use underscores the importance of using a socio-structural determinants framework to understand the vulnerabilities of, and the health risks facing, female sex workers in resource-limited settings. Efforts should be made on policy, community, and local levels to address the overlapping conditions of harmful alcohol use and HIV, and their intersections with these socio-structural factors. The findings of this study strongly support the call from WHO to increase policy and research focus on harmful alcohol use [29]. A particular focus should be directed to this understudied topic among female sex workers.

## Figures and Tables

**Table 1:** Distributions of socio-demographic and socio-structural characteristics of the female sex workers (N = 470).

Variable	*n*	Unweighted Proportion % (95% CI)	Weighted Proportion % (95% CI)
Age (years)			
18–24	199	42.3 (37.9–46.9)	46.3 (39.9–52.8)
25–34	226	48.1 (43.6–52.6)	43.4 (37.2–49.9)
≥35	45	9.6 (7.2–12.6)	10.3 (6.3–16.4)
Marital status			
Never married	361	76.8 (72.8–80.4)	75.8 (69.5–81.2)
Formerly married/currently/married/cohabiting	109	23.2 (19.6–27.2)	24.2 (18.8–30.5)
Highest educational level			
No formal/some primary	28	6.0 (4.1–8.5)	9.7 (5.4–16.9)
Completed primary	165	35.1 (30.9–39.5)	36.9 (31.0–43.3)
Some secondary	138	29.4 (25.4–33.7)	27.2 (21.8–33.4)
Completed secondary or above	139	29.6 (25.6–33.9)	26.1 (21.3–31.6)
Monthly income from sex work * (TZS †)			
<150,000	73	15.9 (12.9–19.6)	19.1 (14.3–-25.0)
150,000–299,999	151	33.0 (28.8–37.4)	28.6 (23.3–34.5)
300,000–449,999	144	31.4 (27.3–35.9)	32.8 (26.6–39.6)
≥450,000	90	19.7 (16.3–23.6)	19.5 (14.7–25.5)
Years since started sex work			
<5	215	45.7 (41.3–50.3)	53.8 (47.2–60.2)
≥5	255	54.3 (49.7–58.7)	46.2 (39.8–52.8)
Number of clients/month (vaginal sex)			
<10	124	26.7 (22.8–30.9)	33.9 (27.7–40.8)
10–29	169	36.3 (32.1–40.8)	35.5 (29.6–42.0)
≥30	172	37.0 (32.7–41.5)	30.5 (25.1–36.6)
Have other work besides sex work			
Yes	187	39.8 (35.4–44.3)	40.6 (34.4–47.1)
No	283	60.2 (55.7–64.6)	59.4 (52.9–65.6)
Female sex worker stigma score ‡			
<30	205	44.9 (40.3–49.5)	44.5 (38.1–51.2)
≥30	252	55.1 (50.5–59.7)	55.5 (50.6–63.6)
Social support score (DUFSS) ^§||^			
≤3	178	38.1 (33.8–42.6)	40.0 (33.5–46.9)
>3	289	61.9 (57.4–66.2)	60.0 (53.1–66.5)
Gender-based violence last 12 months ^¶^			
Yes	241	51.5 (47.0–56.0)	43.7 (37.5–50.2)
No	227	48.5 (44.0–53.0)	56.3 (50.0–62.5)
Arrest/incarceration last 12 months			
Yes	126	26.8 (23.0–31.0)	20.6 (16.1–26.0)
No	344	73.2 (69.0–77.0)	79.4 (74.0–83.9)
Reason for arrest **			
Sex work	75	60.0 (51.1–68.3)	54.7 (41.0–67.6)
Other reasons	50	40.0 (31.7–48.9)	45.4 (32.4–59.0)
Sex work-related mobility last 6 months			
Yes	179	38.1 (33.8–42.6)	28.4 (23.5–33.8)
No	291	61.9 (57.4–66.2)	71.6 (66.2–76.5)
Lifetime drug use			
Yes	64	13.6 (10.8–17.0)	8.3 (6.0–11.4)
No	406	86.4 (83.0–89.2)	91.7 (88.6–94.0)

* 12 missing observations. † 1 USD ≈ 2000 TZS (Tanzanian shillings). ‡ 13 missing observations. ^§^ Duke UNC-functional Social Support Questionnaire (DUFSS). ^||^ Three missing observations; ^¶^ Two missing observations. ** Among those reporting arrest the last 12 months. One missing observation.

**Table 2:** Distributions and patterns of alcohol use among female sex workers (N = 470).

Alcohol Use	*n*	Unweighted Proportion % (95% CI)	Weighted Proportion % (95% CI)
Reported drinking alcohol the past year			
Yes	405	86.2 (82.7–89.0)	82.1 (75.8–87.0)
No	65	13.8 (11.0–17.3)	17.9 (13.0–24.2)
Drinking at last sexual encounter with a client †			
Yes	296	63.1 (58.6–67.4)	53.6 (47.0–60.2)
No	173 *	36.8 (32.6–41.4)	46.4 (39.8–53.0)
Hazardous alcohol use (AUDIT ≥ 8)			
Yes	360	76.6 (72.5–80.2)	69.6 (62.6–75.9)
No	110 *	23.4 (19.8–27.5)	30.4 (24.1–37.4)
Harmful alcohol use (AUDIT ≥ 16)			
Yes	230	48.9 (44.4–53.5)	37.3 (31.7–43.4)
No	240 *	51.1 (46.5–55.6)	62.7 (56.6–68.3)

* Participants who reported not drinking last year are included in “No”-category. † 1 missing observation.

**Table 3:** Distributions of sociodemographic and socio-structural factors by harmful alcohol use (AUDIT scores ≥ 16).

Socio-Demographic and Socio-Structural Factors	N	Harmful Alcohol Use: N (%)	*p*-Value
Age (years)			0.48
18–24	199	92 (46.2)	
25–34	226	113 (50.0)	
≥35	45	25 (55.6)	
Marital status			0.11
Never married	361	184 (51.0)	
Formerly married/currently married/cohabiting	109	46 (42.2)	
Highest educational attainment			0.73
No formal/some primary	28	15 (53.6)	
Completed primary	165	81 (49.1)	
Some secondary	138	71 (51.5)	
Completed secondary or above	139	63 (45.3)	
Monthly income from sex work (TZS *)			0.34
<150,000	73	30 (41.1)	
150,000–299,999	151	75 (49.7)	
300,000–449,999	144	78 (54.2)	
≥450,000	90	44 (48.9)	
Years since started sex work			<0.01
<5	215	86 (40.0)	
≥5	255	144 (56.5)	
Number of clients month (vaginal sex)			0.01
<10	124	46 (37.1)	
10–29	169	88 (52.1)	
≥30	172	93 (54.1)	
Have other work besides sex work			
Yes	187	81 (43.3)	0.05
No	283	149 (52.7)	
Female sex worker stigma score			
<30	205	110 (53.7)	0.11
≥30	252	116 (46.0)	
Social support (DUFSS ^†^)			0.48
≤3	178	91 (51.1)	
>3	289	138 (47.7)	
Gender-based violence last 12 months			<0.01
Yes	241	145 (60.2)	
No	227	84 (37.0)	
Arrest/incarceration last 12 months			<0.01
Yes	126	89 (70.6)	
No	344	141 (41.0)	
Sex work-related mobility last 6 months			<0.01
Yes	179	106 (59.2)	
No	291	124 (42.6)	
Lifetime drug use			<0.01
Yes	64	43 (67.2)	
No	406	187 (46.1)	

* 1 USD ≈ 2000 TZS (Tanzanian shillings). ^†^ Duke UNC-functional Social Support Questionnaire (DUFSS).

**Table 4:** Regression analysis of the association between socio-structural factors and harmful alcohol use.

Independent Variable	Crude PR (95% CI)	Adjusted PR (95% CI)	*p*-Value for APR
Arrest/Incarceration last 12 months *	1.72 (1.45–2.04)	1.55 (1.27–1.84)	<0.01
Gender based-violence last 12 months †	1.63 (1.33–1.98)	1.31 (1.06–1.56)	<0.01
Sex work related mobility last 6 months ‡	1.39 (1.16–1.66)	1.36 (1.11–1.61)	<0.01

* Adjusted for FSW stigma, age, drug use ever, marital status, mobility, number of partners, other job than sex work, years in sex work (n = 452); † Adjusted for FSW stigma, age, drug use ever, incarceration, marital status, mobility, number of partners, other job than sex work, years in sex work (n = 450); ‡ Adjusted for FSW stigma, age, marital status, other work than sex work, years in sex work (n = 457).

## Data Availability

The data from this study can be obtained upon reasonable request by the principal investigator, Elia J. Mmbaga (elia.mmbaga@medisin.uio.no).
